# In vivo acoustic patterning of endothelial cells for tissue vascularization

**DOI:** 10.1038/s41598-023-43299-0

**Published:** 2023-09-26

**Authors:** Eric S. Comeau, Melinda A. Vander Horst, Carol H. Raeman, Sally Z. Child, Denise C. Hocking, Diane Dalecki

**Affiliations:** 1 Department of Biomedical Engineering, University of Rochester, 308 Goergen Hall, P.O. Box 270168, Rochester, NY 14627 USA; 2 Department of Pharmacology and Physiology, University of Rochester, 601 Elmwood Avenue, Box 711, Rochester, NY 14642 USA

**Keywords:** Biomedical engineering, Tissue engineering

## Abstract

Strategies to fabricate microvascular networks that structurally and functionally mimic native microvessels are needed to address a host of clinical conditions associated with tissue ischemia. The objective of this work was to advance a novel ultrasound technology to fabricate complex, functional microvascular networks directly in vivo. Acoustic patterning utilizes forces within an ultrasound standing wave field (USWF) to organize cells or microparticles volumetrically into defined geometric assemblies. A dual-transducer system was developed to generate USWFs site-specifically in vivo through interference of two ultrasound fields. The system rapidly patterned injected cells or microparticles into parallel sheets within collagen hydrogels in vivo. Acoustic patterning of injected endothelial cells within flanks of immunodeficient mice gave rise to perfused microvessels within 7 days of patterning, whereas non-patterned cells did not survive. Thus, externally-applied ultrasound fields guided injected endothelial cells to self-assemble into perfused microvascular networks in vivo. These studies advance acoustic patterning towards in vivo tissue engineering by providing the first proof-of-concept demonstration that non-invasive, ultrasound-mediated cell patterning can be used to fabricate functional microvascular networks directly in vivo.

## Introduction

Most tissues and organs in the body include a dense microcirculatory system that enables gas exchange, provides for nutrient delivery, regulates blood flow, and maintains interstitial fluid balance^1^. Microvascular insufficiency underlies many common, chronic diseases, including peripheral vascular disease, diabetic retinopathy and neuropathy, cerebral infarction, and coronary microvascular disease^2–5^. In vitro vascularization strategies currently being pursued to address microvascular insufficiencies include scaffold functionalization, soft lithography and molding, 3D printing, and modular assembly^6,7^. These approaches face distinct limitations, as they can be time consuming, necessitate extended incubation times and secondary assembly processes, utilize sophisticated equipment and manufacturing processes, have confined geometry and/or limited dimensionality, and cannot be readily reduced to non-invasive, in vivo applications^6^.

Ultrasound technologies offer promising, non-invasive approaches for stimulating both microvascular network formation in vitro and tissue revascularization in vivo. In particular, acoustic patterning techniques provide for rapid, three-dimensional (3D) patterning of cells and/or microparticles, site-specifically and non-invasively^8–10^. Acoustic patterning harnesses radiation forces associated with an ultrasound standing wave field (USWF) to rapidly direct the spatial organization of suspended particles volumetrically^11,12^. An USWF generated between a single incident sound source and an acoustic reflector is characterized by regions of minimum acoustic pressure (pressure nodes) and maximum acoustic pressure (pressure anti-nodes). Nodal planes are oriented perpendicular to the direction of sound propagation and are spaced at intervals equal to one half the wavelength of the incident sound field. Acoustic radiation forces associated with an USWF can be directed either toward or away from the nodal planes depending upon the material properties of the fluid and suspended particles. Specifically, if an aqueous solution of cells or microparticles is placed in an USWF, acoustic radiation forces (*F*_*rad*_) associated with the USWF act on the cells or particles to rapidly drive them to pressure nodal locations^13–15^. The magnitude of *F*_*rad*_ is given as

1 $$F_{rad} = \left( {\frac{{ - \pi P_{o}^{2} V\beta_{o} }}{2\lambda }} \right)* \phi *{\text{sin}}\left( {\frac{4\pi z}{\lambda }} \right)$$

where *P*_*o*_ is the peak pressure amplitude in the USWF, *V* is the volume of the cell, *λ* = *c/f* is the wavelength of the incident sound field, *c* is the speed of sound, *f* is the acoustic frequency of the sound field, and *z* is the perpendicular distance between a cell and the nearest node^14^. The acoustic contrast factor, *Φ*, characterizes the difference in density and compressibility of a cell relative to the surrounding media is defined as

2 $$\phi = \frac{{5\rho_{p} - 2\rho_{o} }}{{2\rho_{p} + \rho_{o} }} - \frac{{\beta_{p} }}{{\beta_{o} }}$$

where, *ρ*_*p*_ and *β*_*p*_ are the density and compressibility of the cell, and *ρ*_*o*_ and *β*_*o*_ are the density and compressibility of the surrounding media^14^. The magnitude of the acoustic radiation force (Eq. 1) is directly proportional to the acoustic contrast factor. Furthermore, when *Φ* is positive, suspended microparticles or cells will be forced towards pressure nodes, and when *Φ* is negative, forces in the USWF will be directed towards pressure anti-nodes. For cells and microparticles used in this study, *Φ* is positive, thus, acoustic radiation forces in the USWF directed cells and microparticles towards pressure nodal planes^16^. The magnitude of the acoustic radiation force in an USWF (Eq. 1) depends on both acoustic parameters and material properties, and various patterning geometries can be achieved using multiple acoustic sources, thus enabling design of spatial patterns.

Acoustic patterning technologies have been employed to organize various cell types within in vitro environments^17,18^. Suspensions of cells can be exposed to an USWF, and acoustic radiation forces pattern cells rapidly and volumetrically. Hydrogels that undergo a phase conversion from liquid to solid during the ultrasound exposure period may be employed such that when the sound is deactivated, the spatial patterning of cells or microparticles is retained^16^. USWF-mediated patterning of endothelial cells within collagen hydrogels in vitro accelerates the emergence of capillary-like sprouts, stimulates collagen fibril alignment, and results in the maturation of lumen-containing, branching vessel networks throughout the collagen hydrogel^8^. The rate of microvessel formation and the morphology of resultant microvascular networks can be influenced by acoustic field parameters employed for patterning^9,10^. In addition to microvascular network formation^8–10,19^, acoustic patterning of other cell types in vitro has been used to stimulate neurite alignment^20^ and neuronal differentiation^21^, fabricate tumor spheroid models^22^, induce myofibrillogenesis^23^, and assemble beating cardiac microtissues^24,25^.

A key advantage of ultrasound is its ability to propagate through tissue as an acoustic beam and exert acoustic radiation forces within tissue non-invasively. Here, we report on investigations to advance acoustic patterning technologies towards in vivo tissue engineering and regenerative medicine. In the present study, a dual-transducer system was developed to generate USWFs site-specifically in vivo through the interference of two ultrasound fields. An USWF formed at the intersection of two propagating ultrasound beams has characteristic nodes and anti-nodes, and the distance between nodal planes, *d*, is given as

3 $$d = \frac{\lambda }{{2{\text{sin}}\left( {\frac{\theta }{2}} \right)}}$$

where λ is the acoustic wavelength of the incident sound fields and θ is the angle of intersection of the two sound fields^20,26,27^. The distance between nodal planes in the USWF can be altered by control of the angle of intersection of the sound fields and acoustic frequency (Eq. 3). Importantly, USWFs are generated only where ultrasound fields intersect.

Investigations in this paper utilized the dual-transducer system to pattern cells and microparticles in mice in vivo, site-specifically and non-invasively. To provide initial evidence for feasibility of clinical translation, the ability to generate USWF through tissue and control tissue heating during acoustic patterning were demonstrated. In vivo acoustic patterning of endothelial cells resulted in the de novo formation of perfused microvascular networks, providing the first proof-of-concept that non-invasive, ultrasound-mediated patterning methods can be used to fabricate functional microvascular networks directly in vivo.

## Results

### Acoustic patterning in vitro using an USWF dual-transducer system

**Figure 1:**
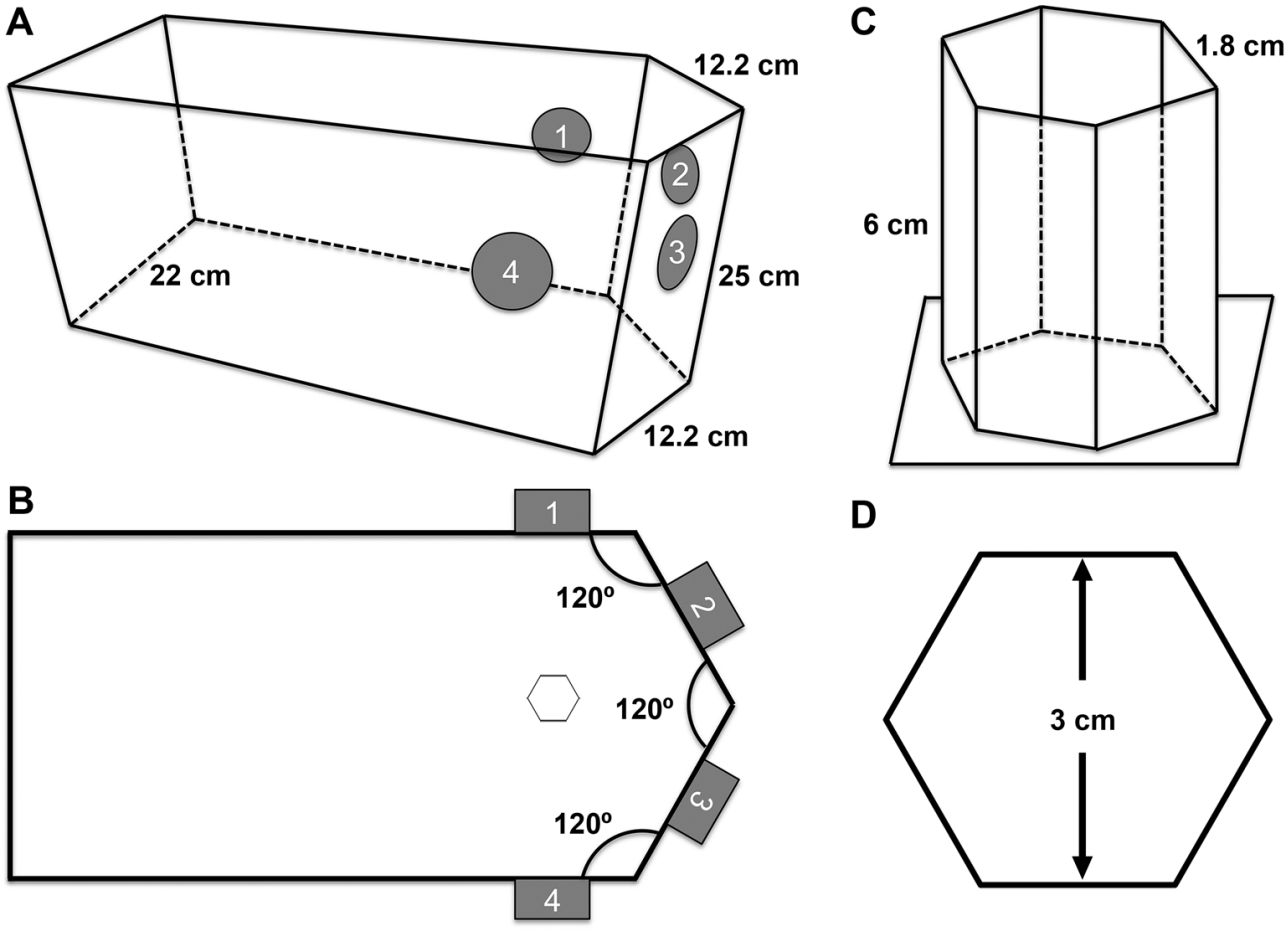
Schematics of dual-transducer USWF system and sample holder. (**A**,**B**) A plastic tank was constructed to hold ultrasound transducers at four locations (sites 1–4). Activation of two transducers simultaneously will produce an USWF at the location of the intersecting beams. (**A**) 3D and (**B**) top-down illustrations of the tank are shown. (**C**,**D**) Hexagonal sample holders with dimensions of 1.2 × 6 × 0.2 cm (w × l × t) were constructed from TPX plastic. (**C**) 3D and (**D**) top-down illustrations are shown. Location of the sample holder within the exposure tank is shown in (**B**).

A custom dual-transducer system was developed to generate USWFs used for all acoustic patterning experiments. A pentagonal, plastic tank was fabricated to position ultrasound transducers at defined angles for the generation of USWFs (Fig. 1A,B). Piezoceramic, 1-MHz, 1-inch diameter, unfocused transducers were inserted into the sides of the tank such that when two transducers were activated simultaneously, an USWF was generated at the location of the intersecting sound beams. Here, angles of intersection of beams from discrete pairs of transducers were 60°, 120°, or 180°. Transducers were positioned such that the last axial maximum of each sound field co-aligned at a point equidistant from each transducer (10.75 cm). Hexagonal sample holders for in vitro experiments (1.2 × 6 × 0.2 cm, w × l × t; Fig. 1C,D) were fabricated from TPX plastic (Mitsui Chemicals, Rye Brook, NY), a material that is nearly acoustically transparent and non-reflective at a frequency of 1 MHz^28^. The sample holder was positioned using a three-way positioner such that the center of the holder was located at the intersection of the last axial maxima of the transducers (Fig. 1B).

**Figure 2:**
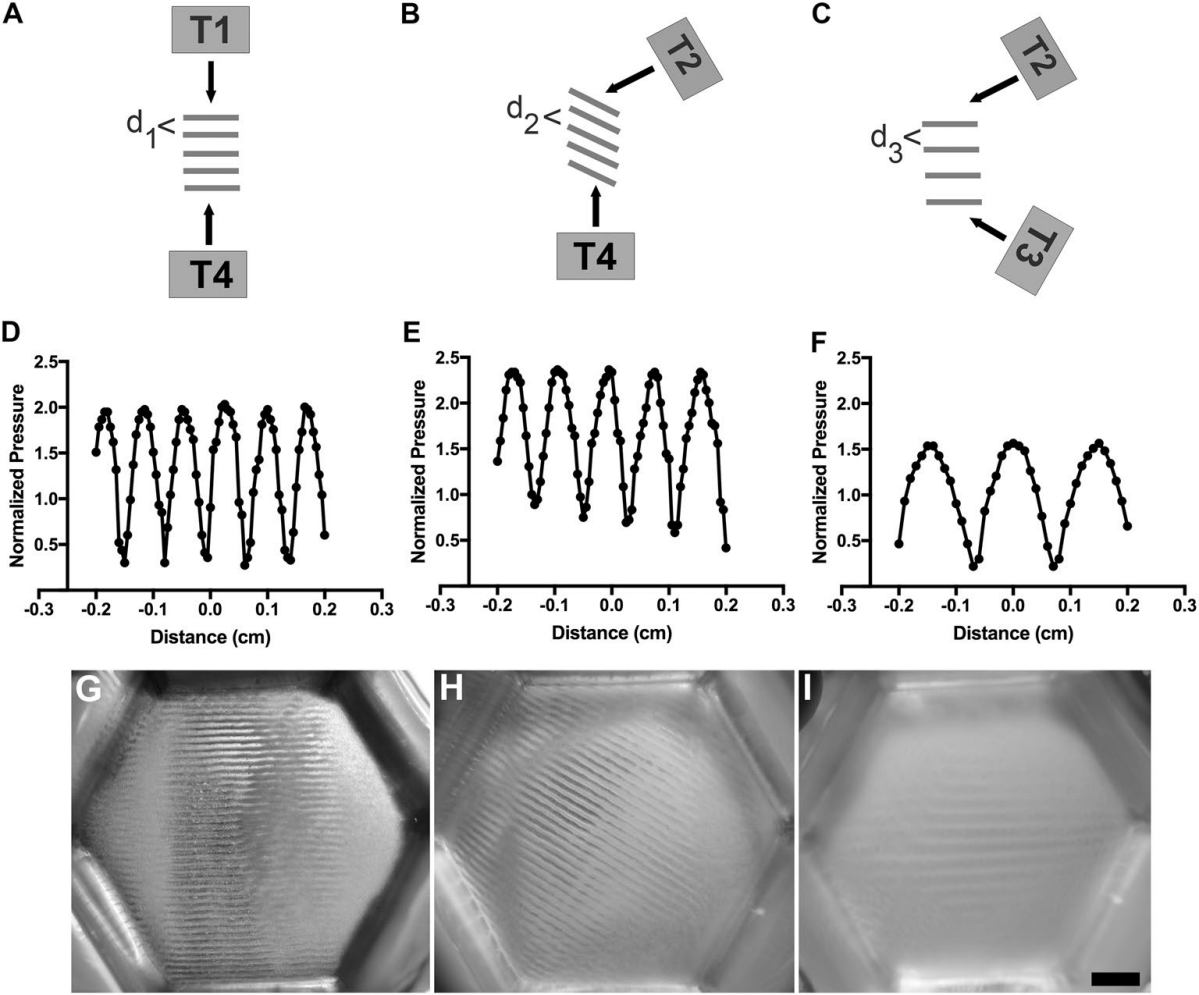
USWF-patterning of microparticles in vitro. (**A**–**C**) An USWF is generated at the intersection of two ultrasound fields. The angle of intersection of the fields influences the spacing and orientation of nodal planes (Eq. 3). Illustrated are beams intersecting at 180° (**A**), 120° (**B**), and 60° (**C**). ‘T’ denotes placement of transducers. (**D**–**F**) A membrane hydrophone was used to measure axial distributions of pressure in an USWF for angles of 180° (**D**), 120° (**E**), and 60° (**F**). Pressure measurements were normalized to the average of the free-field pressure measured from each active transducer. (**G**–**I**) Sephadex particles (10–40 µm diameter) were suspended in 1% (w/v) agarose solutions and exposed to an USWF generated by two transducers. Shown are images of microparticle patterning using 1-MHz ultrasound beams intersecting at 180° (**G**), 120° (**H**) and 60° (**I**). Images are representative of n = 3–4 gels fabricated over 4 independent experiments. Scale bar = 5 mm.

The dual-transducer USWF exposure system was first evaluated for microparticle patterning in vitro. Microparticles (Sephadex G-25; 10–40 µm) were used in place of cells for initial design and evaluation experiments to facilitate rapid, iterative system development. As illustrated in Fig. 2A, simultaneous activation of two transducers results in the generation of an USWF at the location of the intersecting ultrasound beams. Referring to Fig. 1B, simultaneous activation of transducers (i) 1 and 2 (or alternatively, 3 and 4, or 2 and 3) produces beams intersecting at 60°; (ii) 1 and 3 (or 2 and 4) produces beams intersecting at 120°, and (iii) 1 and 4 produces beams intersecting at 180°. The angle between the beams from two activated transducers influences the spacing of the nodal planes (*d*) of the USWF as described by Eq. (3), and the nodal planes align along the vector that bisects the angle made by the propagating ultrasound fields from the two transducers, as depicted in Fig. 2A–C^26^. Theoretical predictions of distances between nodal planes for an USWF generated with 1-MHz transducers with beams intersecting at angles of 180°, 120°, and 60° are 750 µm, 866 µm, and 1500 µm, respectively, assuming a speed of sound of 1500 m/s (Eq. 3). Axial spatial distributions of pressure generated by the USWF dual-transducer system with transducer beams intersecting at 180°, 120°, and 60° were measured with a membrane hydrophone. USWFs, comprised of characteristic pressure nodes and antinodes, were observed for all three transducer orientations tested and distances between pressure nodes increased with decreasing angle between transducers (Fig. 2D–F), consistent with Eq. (3).

Sephadex particles suspended in 1% agarose solutions were placed in the custom hexagonal sample holder and exposed to USWFs generated by two transducers with beams intersecting at angles of 180°, 120°, or 60° (Fig. 1B). Upon activation of transducers, acoustic radiation forces associated with the USWF rapidly (within seconds) drove microparticles to nodal planes. Agarose solutions solidified during the 10-min exposure, thereby maintaining particle patterning after transducers were deactivated. Figure 2 provides top view images of hydrogels after USWF exposure and demonstrates microparticle patterning into planar bands using transducers with beams oriented at 180° (Fig. 2G), 120° (Fig. 2H), and 60° (Fig. 2I). Spacing of microparticle bands increased with decreasing transducer angle and was consistent with both theoretical predictions (Eq. 3) and measured USWF beam patterns. Measured distances between bands of microparticles were 766 ± 6.8 µm, 823 ± 2.7 µm, and 1483 ± 7.2 µm (mean ± SEM, n = 3–4) for angles of 180°, 120°, and 60°, respectively. The orientation of the microparticle bands (Fig. 2G–I) was also consistent with the theory^26^ that USWF nodes align along the vector that bisects the angle made by the propagating ultrasound fields from the two transducers. These data demonstrate the feasibility of using USWFs generated by a dual-transducer system to pattern microparticles non-invasively with control over the orientation, location, and spacing of the resulting planar bands of microparticles.

### Acoustic patterning through intervening tissue

**Figure 3:**
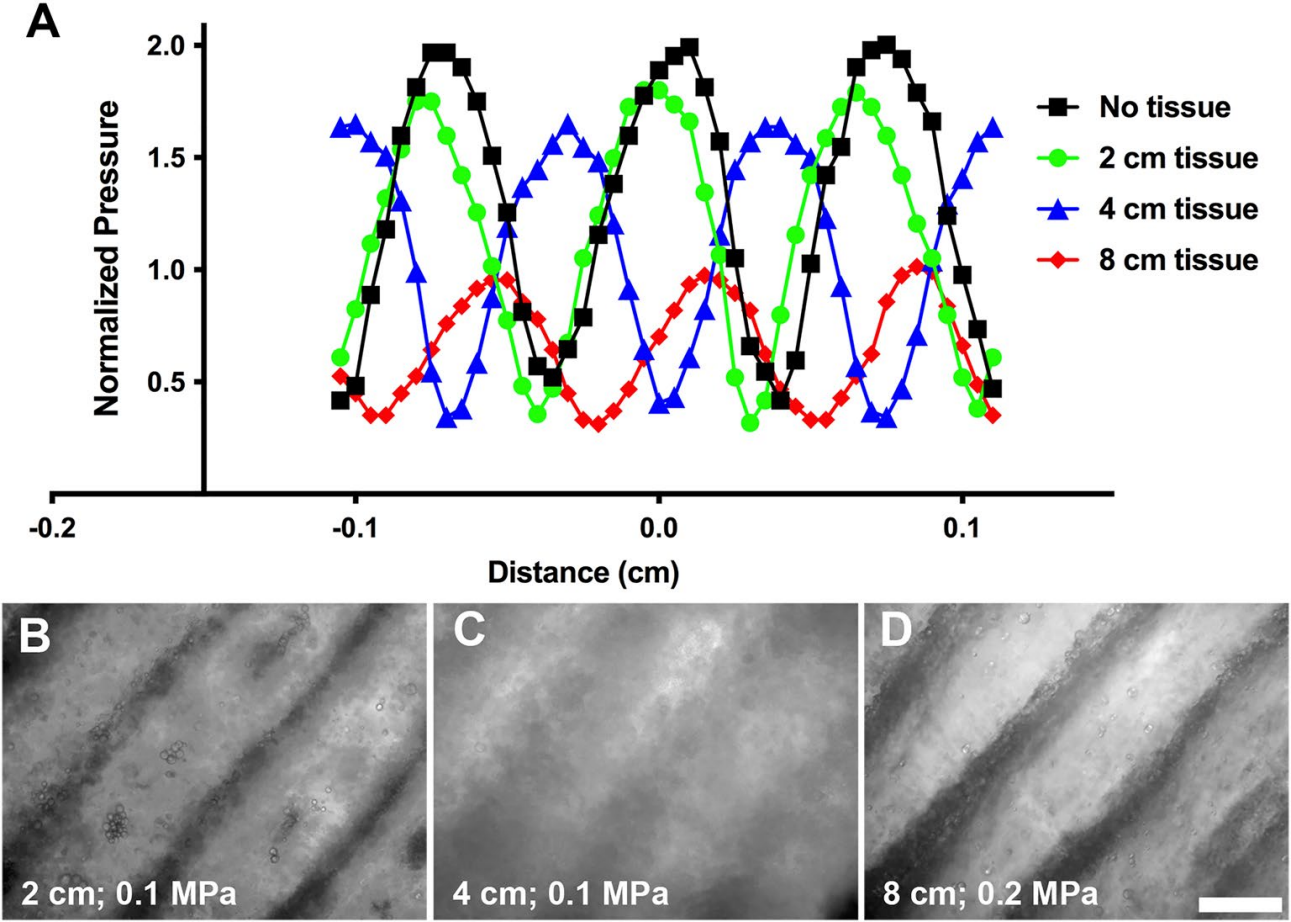
USWF formation and acoustic patterning through intervening tissue. (**A**) Hydrophone measurements were used to determine axial spatial distributions of pressure in the free field in the presence of no tissue (black squares), or 2 cm (green circles), 4 cm (blue triangles), or 8 cm (red diamonds) of total intervening porcine muscle tissue. USWF pressure measurements were normalized to the average free field pressure measured from each active transducer. (**B**–**D**) Sephadex particles (10–40 µm diameter) suspended in collagen solutions (2 mg/mL) were exposed to 1-MHz USWFs at 0.1 (**B**,**C**) or 0.2 (**D**) MPa for 15 min. Collagen solutions polymerized during USWF exposure, thereby maintaining particle patterning after sound deactivation. Shown are representative microscopy images of Sephadex particles (phase dense) patterned using 1-MHz ultrasound beams intersecting at 180° in the presence of 2 (**B**), 4 (**C**), or 8 (**D**) cm of total intervening tissue. Images are representative of 3 gels per exposure condition, fabricated over 3 independent experiments. Scale bar = 500 µm.

Ultrasound attenuation in tissue results in an exponential decrease of acoustic pressure amplitude with distance of propagation. Thus, experiments were performed to determine the influence of intervening tissue on acoustic patterning. Using the dual transducer system, a membrane hydrophone was placed at the midpoint of 2 transducers operating in the 180° configuration. Porcine muscle samples were positioned on each side of the membrane hydrophone and tissue thicknesses of 1, 2, and 4 cm were tested (giving total tissue thicknesses of 2, 4, or 8 cm). Axial distributions of pressure were measured with the hydrophone. USWFs were observed for all tissue thicknesses (Fig. 3A). As expected, maximum pressure amplitudes of the USWF were attenuated with increasing thickness of intervening tissue (Fig. 3A). However, evenly spaced pressure nodes and anti-nodes characteristic of an USWF were still observed in the presence of up to 8 cm of total intervening tissue (Fig. 3A). The full width at half maximum was approximately 500 µm for all conditions (Fig. 3A). The shift in the location of peak amplitudes occurs due to differences in speed of sound in tissue and water.

The reduction in pressure amplitude from the presence of intervening tissue also reduces the magnitude of the acoustic radiation force associated with the USWF (Eqs. 1, 2). Thus, the ability to acoustically pattern Sephadex particles in soluble collagen through intervening porcine tissue was tested using 2 transducers operating at 180°. Porcine muscle tissue with thicknesses of 1, 2, or 4 cm were placed on either side of the cuvette. Samples were exposed to USWFs with maximum pressure amplitude of 0.1 MPa or 0.2 MPa for 15 min. Phase contrast microscopy images of gels revealed that microparticle patterning was present in the absence and presence of intervening tissue up to 8 cm total thickness (Fig. 3B–D). As expected, higher pressure amplitude was required to pattern through intervening tissue efficiently (Fig. 3B–D). In previous work, we showed that decreasing pressure amplitude reduced patterning efficiency, resulting in an increase in cell band width at nodal planes^10^. Comparison of Fig. 3B,C illustrates a similar reduction in patterning efficiency resulting from a decrease in pressure amplitude due to attenuation. Importantly, these data demonstrate that acoustic patterning can be achieved through intervening tissue using appropriate exposure amplitudes.

### Acoustic patterning of microparticles in vivo

**Figure 4:**
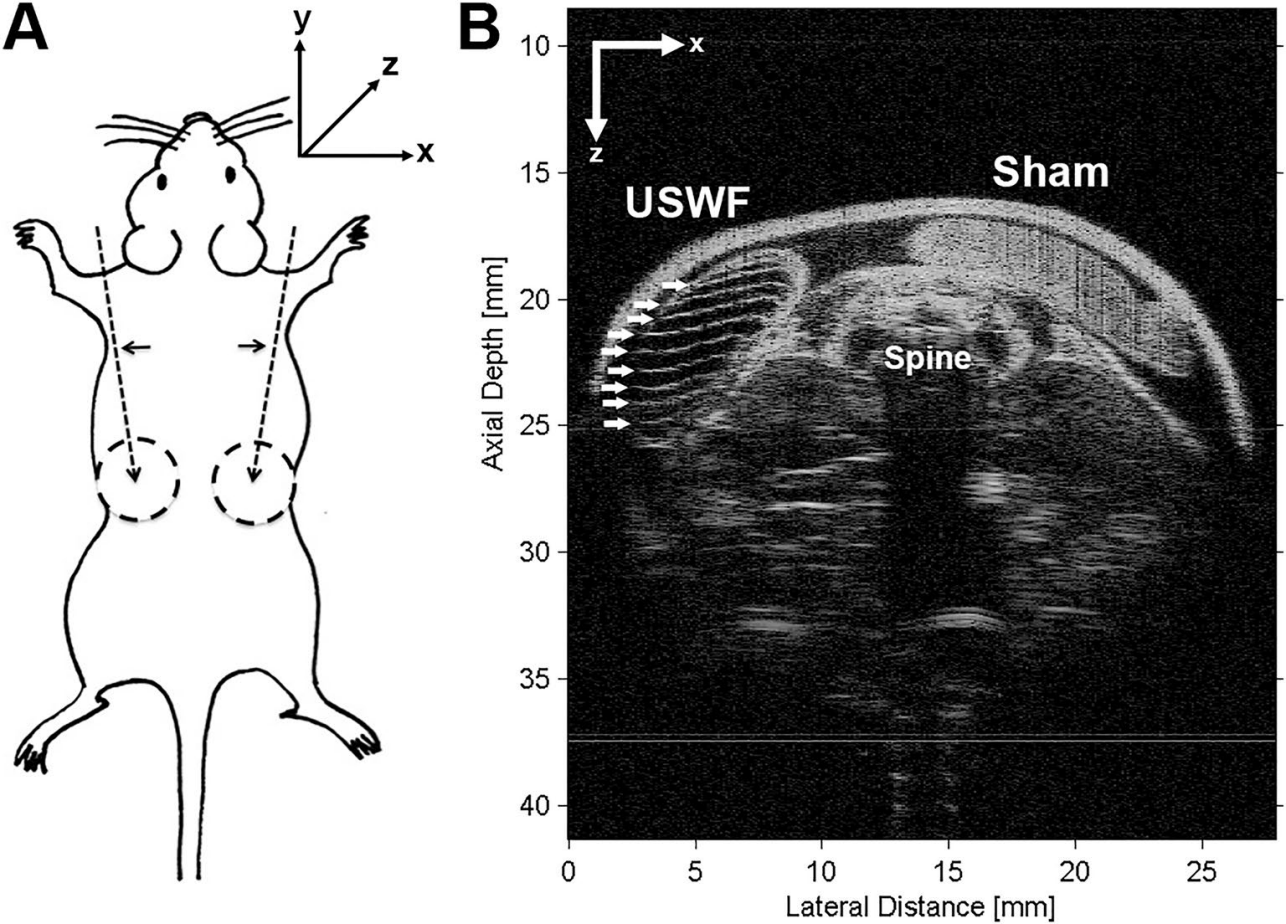
In vivo acoustic patterning of collagen-suspended microparticles in mice. (**A**) Schematic of experimental approach. Needles (dashed arrows) were inserted below the shoulders (horizontal arrows). Collagen-cornstarch solutions (500 µL) were injected into preformed dorsal skin pockets (dashed circles) on either side of the mouse, and one site was exposed to an USWF. The other injection site was located on the contralateral side, outside of the acoustic field. (**B**) Representative high-frequency ultrasound image of a cross-sectional plane of a mouse post-exposure. In this example, the USWF was generated with transducers oriented at 180° with peak pressure of 0.3 MPa (25% duty factor, 560 µs pulse duration) for 10 min. The injection site exposed to the USWF was characterized by evenly spaced, echogenic bands of cornstarch particles (white arrows) beneath the skin. The sham-exposed injection site was characterized by homogeneous echogenicity indicative of a random distribution of cornstarch particles.

Studies were performed next to test the ability of the USWF dual-transducer system to pattern microparticles in vivo. Mice (C57BLKS/J) were anesthetized and prepared for USWF exposure as described in “Materials and methods”. Collagen and cornstarch (2–30 μm diameter^29^) solutions were injected into two preformed subcutaneous pockets on the dorsal flanks of mice (illustrated in Fig. 4A). A three-axis positioner was used to place one injection site within the USWF generated by the dual-transducer system (similar to the location of the sample holder shown in Fig. 1B). The other injection site was located outside of the sound field and served as a control. USWFs were produced with beams intersecting at different angles, with pressure amplitudes of 0.2 or 0.3 MPa, and with either continuous wave (c.w.) or pulsed modes (25% duty factor, 560 µs pulse duration). After USWF exposure, mice were sacrificed and high-frequency ultrasound imaging was utilized to visualize hydrogels beneath the skin.

Figure 4B shows a representative cross-sectional B-scan image of a mouse imaged with high-frequency ultrasound following USWF exposure. In this example, USWFs were generated with two transducers oriented at 180° and peak pressure of 0.3 MPa (25% duty cycle, 560 µs pulse duration). Both hydrogels were clearly evident beneath the skin surface. The B-scan image of the USWF-exposed hydrogel showed bright, echogenic bands of cornstarch granules (Fig. 4B, left side). As seen in the B-scan, the spacing of parallel cornstarch bands within the gel was approximately 7 bands over 5 mm, consistent with the expected nodal distance of 750 µm (Eq. 3). In contrast, the B-scan image of the corresponding sham-exposed injection site showed homogenous echogenicity, indicative of randomly dispersed cornstarch scatterers in the collagen gel (Fig. 4B, right side). Additional examples of in vivo acoustic patterning using USWFs produced with beams intersecting at different angles, with pressure amplitudes of 0.2 or 0.3 MPa, and with either c.w. or pulsed modes are provided in Supplemental Fig. 1. Acoustic patterning was achieved with cornstarch or Sephadex particles, and using either c.w. or pulsed modes. As predicted (Eq. 3), decreasing the transducer angle increased the distance between microparticle bands (sFig. 1). Taken together, these results demonstrate the feasibility of using USWFs to site-specifically pattern microparticles in vivo with control over particle band spacing.

### Acoustic patterning of endothelial cells in vivo

**Figure 5:**
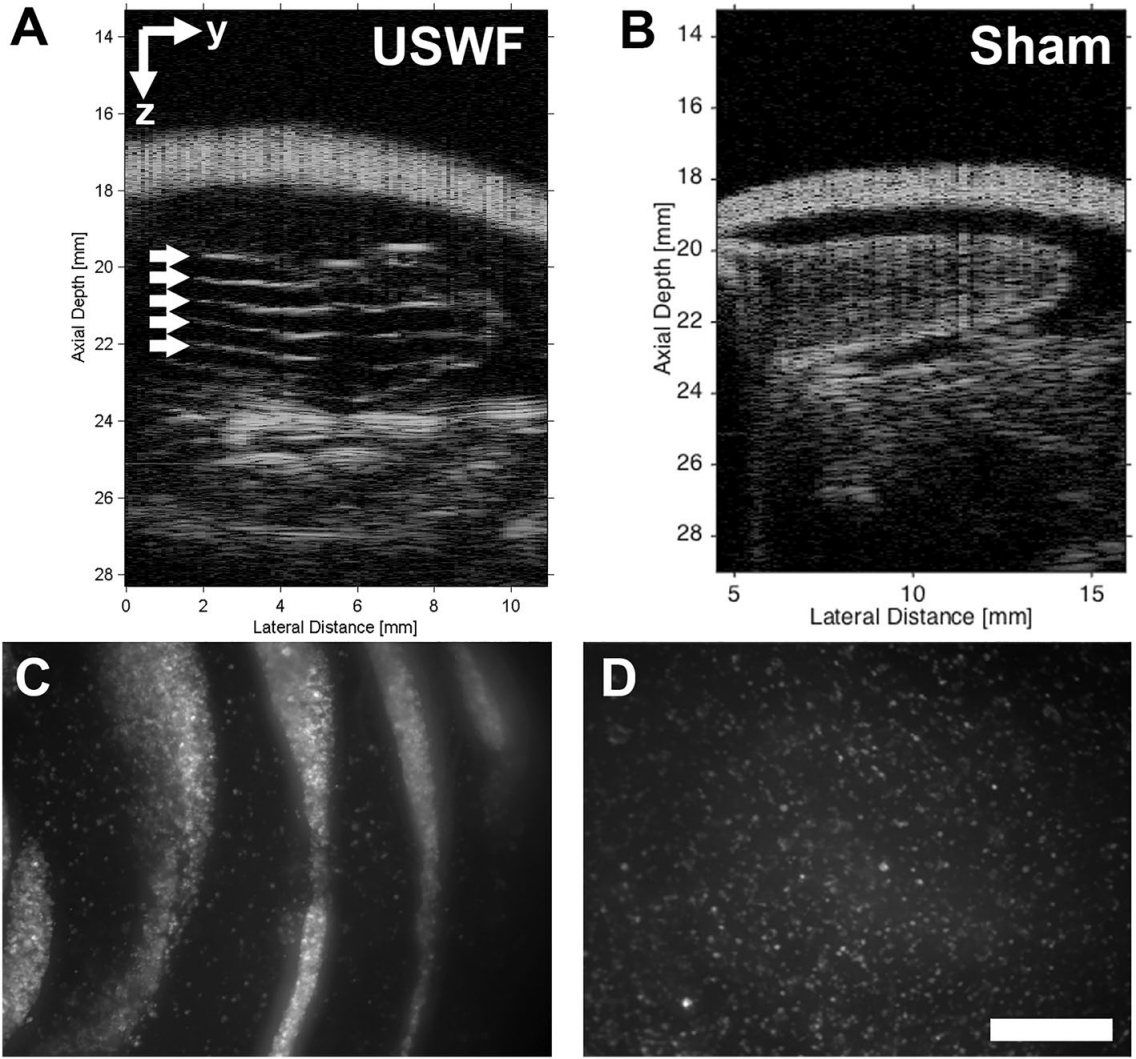
In vivo acoustic patterning of collagen-suspended GFP-HUVECS in mice. GFP-HUVECs (1 × 10^6^ c/mL) were suspended in collagen (1 mg/mL), injected into 2 preformed dorsal skin pockets (0.5 mL/site) of C57BLKS/J mice, and then exposed to either USWF (0.3 MPa, c.w., 10 min, 180°) or sham conditions. (**A**,**B**) Representative high-frequency ultrasound images of a cross-sectional plane of a mouse post-USWF exposure showing (**A**) USWF- and (**B**) sham-exposed hydrogels under the skin. Evenly-spaced planes of GFP-HUVECs are denoted by arrows. (**C**,**D**) Hydrogels were removed from the mouse post-exposure and examined by confocal fluorescence microscopy. Representative z-stack images of (**C**) USWF-exposed, and (**D**) sham-exposed hydrogels. GFP-HUVECs appear white. Images are representative of 5 mice exposed on 4 experimental days. Scale bar = 500 µm.

To assess the ability of USWFs to pattern cells in vivo, green fluorescence protein-expressing HUVECs (GFP-HUVECs) and collagen solutions were delivered into preformed, subcutaneous dorsal pockets. One injection site was exposed to an USWF using the dual-transducer system for 10 min; the other site served as the sham-exposure control. After exposure, mice were imaged using high-frequency ultrasound. Figure 5 provides an example of USWF-pattering of cells within a collagen hydrogel in vivo using an USWF pressure amplitude of 0.3 MPa, using the180° configuration. Similar to results obtained with collagen-suspended microparticles (Fig. 4B), B-scan images showed echogenic, evenly spaced planar bands of cells only at sites exposed to USWFs (white arrows, Fig. 5A). Cell bands were spaced at a distance of 718 ± 51 µm (mean ± SEM, n = 5 mice), consistent with predicted nodal spacing of 750 µm (Eq. 3). B-scan images of sham-exposed collagen-HUVEC gels exhibited homogenous echogenicity with no evidence of planar cell banding (Fig. 5B). As further confirmation of cell patterning, gels were excised and visualized using fluorescence confocal microscopy. Discrete bands of GFP-HUVECs were observed in gels excised from USWF-exposed injection sites (Fig. 5C), whereas GFP-HUVECs were distributed homogenously in sham-exposed gels (Fig. 5D). These results indicate that the dual-transducer USWF system can site-specifically pattern cells administered in collagen solutions in vivo.

**Figure 6:**
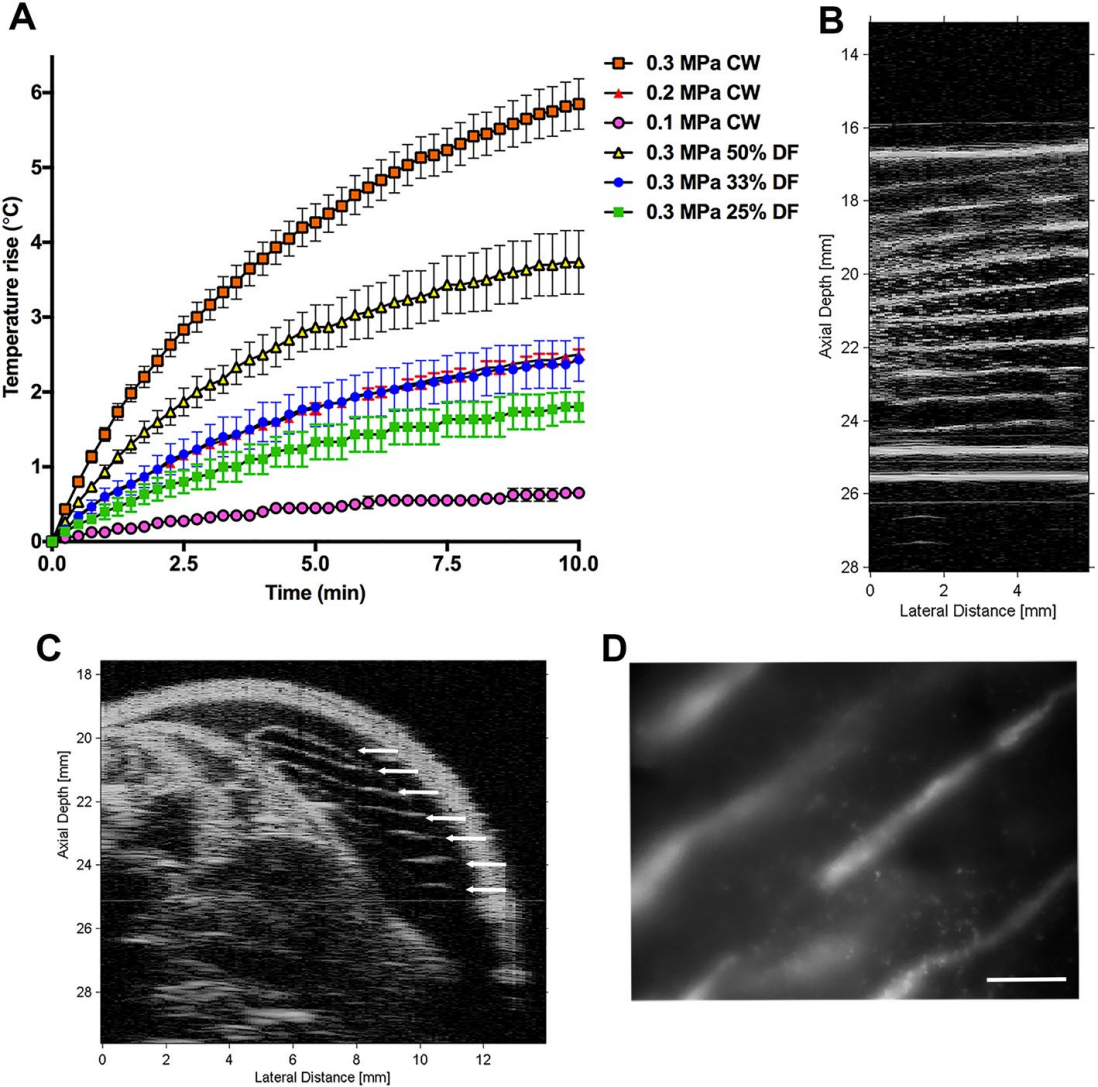
Rapid acoustic patterning of endothelial cells in vivo. (**A**) Heating profiles of porcine tissue exposed to USWFs ex vivo. Temperature rise was measured every 15 s during a 10-min exposure to 0.3 MPa, c.w. (red squares), 0.2 MPa, c.w. (red triangles), 0.1 MPa, c.w. (pink circles), or 0.3 MPa USWFs with 50% (yellow triangles), 33% (blue circles), or 25% (green squares) duty factor. Data represent mean ± SEM of 3–6 measurements taken on 5 independent experimental days. (**B**) Sephadex microparticles were suspended in 1 mg/mL collagen and exposed in vitro to USWFs (1-MHz, c.w., 0.3 MPa, 180°) for 3 min. Representative high frequency ultrasound image of 1 of 4 fabricated gels. (**C**,**D**) Collagen-suspended GFP-HUVECs (1 × 10^6^ c/mL) were injected into preformed dorsal skin pockets of C57BLKS/J mice, and exposed to USWF (0.3 MPa, c.w., 180°) for 3 min. Mice remained in the warm water tank for an additional 7 min to allow for collagen polymerization. Representative high-frequency ultrasound image of a cross-sectional plane of a mouse post-exposure showing the USWF-exposed hydrogel. Evenly-spaced planes of GFP-HUVECs are denoted by arrows. (**D**) Hydrogels were extracted from the mouse post-exposure and examined by confocal fluorescence microscopy. Representative z-stack image of USWF-exposed hydrogel showing cross-section of planar bands of GFP-HUVECs (white). Scale bar = 500 µm. Images are representative of 4 mice exposed on 4 independent experimental days.

As ultrasound propagates through tissue, a portion of the ultrasound energy is absorbed by the tissue and converted to heat. The ability to control tissue heating during acoustic patterning is a necessary step towards clinical translation. Thus, to evaluate heating associated with acoustic patterning, porcine muscle tissue samples were exposed to USWFs and temperatures were measured. Experiments were performed with tissues ex vivo to eliminate cooling effects from blood flow around the exposure site and provide worst case estimates. Ten minutes of c.w. exposure (1 MHz) to an USWF with peak amplitude of 0.3 MPa, 0.2 MPa, or 0.1 MPa resulted in average temperature increases of 5.8 ± 0.34 °C, 2.5 ± 0.07 °C, and 0.65 ± 0.06 °C, respectively (Fig. 6A). Pulsed ultrasound exposures with peak amplitude of 0.3 MPa resulted in temperature increases of 3.7 ± 0.42 °C, 2.4 ± 0.29 °C, and 1.8 ± 0.20 °C for duty factors of 50%, 33%, 25%, respectively (Fig. 6A). The American Institute of Ultrasound in Medicine (AIUM) and other scientific organizations have provided thermal safety guidelines for ultrasound^30^. An empirical equation to provide guidance on combinations of temperature rise and maximum exposure duration without adverse effect in post-natal mammalian tissue is

4 $$\Delta {\text{T }} < { 6}\,^{\circ}{\text{C }}- \left( {{\text{C}}_{{\text{T}}} /0.6} \right)\left( {{\text{log}}_{{10}}\, \left( {{\text{t}}/{\text{t}}_{{\text{c}}} } \right)} \right)$$

where ΔT is the change in temperature (°C), t is the exposure time (min), C_T_ = 1 °C, and t_c_ = 1 min^31^. Thus, for USWF durations of 10 and 3 min, recommended temperature increases should not exceed 4.3 °C and 5.2 °C, respectively.

Reduction in ultrasound-induced tissue heating can be achieved by reducing the total exposure time, reducing the peak pressure amplitude, or by using pulsed exposures. Results shown in Fig. 6A indicate that a 3-min exposure with peak amplitude of 0.3 MPa produced a maximum temperature rise of 3.2 ± 0.20 °C, which is within thermal safety guidelines^30^. In vitro studies confirmed that acoustic patterning occurred within 3 min (Fig. 6B). Thus, we next investigated acoustic cell patterning in vivo using the shorter exposure duration of 3 min. High-frequency ultrasound imaging of collagen-HUVEC injection sites exposed to USWFs for 3 min revealed evenly spaced (700 ± 55 µm, n = 3) echogenic bands of cells (Fig. 6C). Cell patterning was not observed in sham-exposed gels (not shown). Subsequent confocal fluorescence imaging of excised gels confirmed that GFP-HUVECs were patterned in response to 3-min exposures to USWFs (Fig. 6D).

### Microvessel formation in response to acoustic patterning in vivo

**Figure 7:**
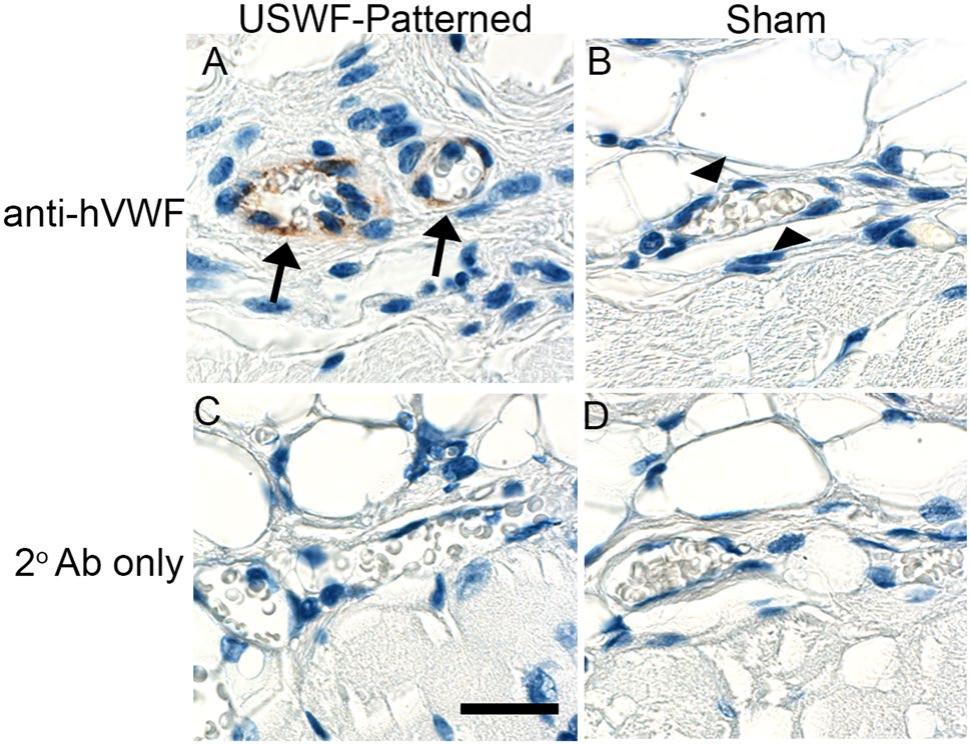
Formation of functional microvessels in mice following in vivo acoustic patterning of endothelial cells. Solutions (0.5 mL) of HUVECs (1 × 10^6^ cells/mL) and collagen (1 mg/mL) were injected into preformed subcutaneous pockets located on the flanks of Rag1^null^ mice. One site was exposed to an USWF (1 MHz with peak amplitude of 0.3 MPa, c.w.) for 3 min. Seven days after acoustic patterning, mice were sacrificed and tissues corresponding to the injection sites were removed and processed for IHC. Human-derived endothelial cells were identified using an antibody specific to human VWF. Shown are representative images from 1 of 6 mice tested. (**A**) hVWF-positive blood vessels (arrows) containing red blood cells were observed in USWF-exposed tissues. (**B**) Only hVWF-negative blood vessels (arrow heads) were observed in sham-exposed tissues. No staining was observed in sections incubated with secondary antibodies only (**C**,**D**). Scale bar = 25 mm.

In previous work^8,10^, we demonstrated that USWF-patterning of endothelial cells in collagen hydrogels in vitro stimulated the formation of three-dimensional, lumen-containing microvascular networks. Thus, we next asked whether acoustic patterning of endothelial cells in vivo could lead to site-specific generation of new, blood-perfused microvessels. Immunodeficient Rag1^null^ mice were anesthetized and prepared for exposure as described in “Materials and methods”. GFP-HUVECs and collagen solutions were delivered to the dorsal flanks. One injection site was exposed for 3 min to an USWF with peak pressure amplitude of 0.3 MPa using the dual-transducer system configured at 180°. Exposures of 0.3 MPa for 3 min were used as these conditions produced acoustic patterning with minimized tissue heating (Fig. 6A). Mice were sacrificed 7 days after acoustic exposures, and sham- and USWF-exposure sites and surrounding tissue were excised, fixed, and processed for immunohistochemistry. HUVECs were identified using a species-specific antibody to human von Willibrand factor (hVWF). Representative images of USWF-patterned and sham-exposed tissues are shown in Fig. 7 and Supplemental Fig. 2. hVWF-positive blood vessels that contained red blood cells were observed in tissue sections from USWF-exposed areas (Fig. 7A), indicating that USWF-patterned HUVECs formed new microvascular networks that had anastomosed with host blood vessels. hVWF-positive blood vessels were clearly present in resected, USWF-exposed tissues from 5 of 6 mice evaluated. In contrast, hVWF staining was not observed within sham-exposed areas (Fig. 7B), indicating the absence of viable HUVECs 7 days post-injection. No staining was observed in tissue sections incubated with secondary antibodies only (Fig. 7C,D). Furthermore, hVWF-positive and hVWF-negative blood vessels were observed within the same tissue section (sFig. 2), providing additional evidence of staining specificity.

## Discussion

In this paper, a dual-transducer system was designed and fabricated to generate USWFs at the location of two intersecting ultrasound beams. Initial tests in vitro demonstrated the utility of this system for patterning microparticles within collagen hydrogels. The dual-transducer system, combined with a cell/hydrogel injection protocol, was then utilized to rapidly and site-specifically pattern endothelial cells within collagen hydrogels in mice in vivo. High-frequency ultrasound imaging visualized and confirmed acoustic patterning, while microscopic analyses of resected hydrogels provided further confirmation of patterned endothelial cells. In vivo acoustic patterning of GFP-HUVECs within the flanks of immunodeficient Rag1^null^ mice gave rise to perfused microvessel networks within 7 days of initial acoustic patterning.

Acoustic patterning in vivo relies on radiation forces and characteristics of an USWF. In this work, USWFs were generated using a dual-transducer system consisting of two ultrasound transducers oriented with intersecting propagation paths such that a standing wave field was generated within the volume of the intersecting beams. The acoustic field formed from this geometry is characterized by planar locations of pressure nodes and antinodes, and the distance between the nodal planes can be designed by choice of acoustic frequency and angle between the transducers. As shown in Fig. 2, changing the angle of intersection of the ultrasound fields allowed for control of the distance between nodal planes, and thus, control over the spacing of aggregates. Previous work from our lab showed that initial spacing between planar bands of USWF-patterned endothelial cells influenced the morphology of the resulting microvascular network, particularly its branching characteristics^10^. In those studies, spacing of planar bands was controlled by changing ultrasound transducer frequency^10^. Utilizing a dual-transducer system for cell patterning provides flexibility to control nodal spacing and thus, particle or cell patterning, by changing the angle of beam intersection without the need to change the frequency of the transducer.

The magnitude of the acoustic radiation force in an USWF is dependent upon both acoustic parameters and material properties, thereby providing opportunity to design exposure conditions to control spatial patterning. In general, USWFs are produced by the interference of two or more ultrasound fields. In the current study, the dual-transducer system was designed to produce planes of nodes and anti-nodes in the USWF, resulting in formation of planar bands of microparticles or cells. More complex spatial patterns of cells or microparticles can be achieved by designs that incorporate two or more acoustic sources and/or reflectors in various geometric orientations. Our previous work provided evidence that acoustic fields can be designed to pattern particles in planes, columns, and grids^32^. Furthermore, particle patterning in vitro has been demonstrated using a single transducer combined with a holographic lens to generate unique patterns through design of the acoustic hologram^33–35^.

A unique advantage of ultrasound technologies is the ability to generate site-specific acoustic fields and associated acoustic radiation forces in tissues, non-invasively. Studies reported in this paper advance acoustic patterning techniques by translating this technology to site-specific in vivo patterning. The dual-transducer system, combined with a cell/hydrogel injection protocol, produced rapid patterning of cells and microparticles in mice in vivo, and this patterning was confirmed with both high-frequency ultrasound imaging and microscopic visualization of dissected hydrogels (Figs. 4, 5, 6 and sFig. 1). These studies were performed in mice, but potential use of the technology with larger animals is supported by demonstration of the ability to generate USWFs and associated particle patterning through thicker overlying tissue layers (Fig. 3). Furthermore, investigations of ultrasound-induced heating confirmed that acoustic pattering in vivo can be achieved by minimizing heating through proper design of acoustic exposure parameters including exposure duration and pulsing parameters (Fig. 6). Here, endothelial cells were used for in vivo acoustic patterning. However, acoustic patterning techniques are expected to have broad applicability to other cell types^20–25^. As well, acoustic patterning of microparticles in vivo may offer innovative avenues for tissue engineering and regenerative medicine, particularly if the microparticles include bound bioactive molecules that provide cues to initiate cellular behaviors^16^. Acoustic vaporization techniques^36^ for controlled release of growth factors that influence angiogenesis may also provide a complementary approach for tissue revascularization.

Investigations in this report present a novel dual-transducer system for generating USWFs in vivo and provide the first demonstrations that in vivo acoustic patterning of endothelial cells within injected hydrogels leads to site-specific microvessel network formation. Continued investigations will expand on the limitations of the current studies and extend the potential scope of applications of this technology. Previous in vitro studies from our lab^10^ demonstrated that the morphology of resultant microvessels can be influenced by acoustic parameters used for initial spatial patterning of endothelial cells. Future studies are needed to test whether spatial geometries of acoustically-patterned cells similarly influence microvessel network formation and morphology in vivo. While work in this report has demonstrated feasibility of generating USWFs through thicker intervening tissue and the ability to control heating through acoustic exposure parameters, additional advances are needed to move acoustic patterning towards clinical translation including, innovative instrumentation systems for generating USWFs in vivo for specific clinical targets, refinement of hydrogel formulations, measurements of resultant tissue perfusion, and real-time image guidance techniques.

In summary, work reported herein offers a novel ultrasound technology that provides a non-invasive patterning tool to site-specifically stimulate microvessel network formation within injected hydrogels in vivo. Creating microvessel networks that structurally and functionally mimic the native microvasculature of various tissues is critical for a wide range of tissue engineering and regenerative medicine applications. The use of USWF for in vivo patterning and microvascular engineering is ideal as it is non-invasive, rapid (< 5 min), inexpensive, effective with both cells and microparticles, can be adapted to various dimensions, does not affect cell viability, and can propagate through tissue as a focused beam. Ultrasound field parameters are highly controllable, thus allowing design of optimized exposure scenarios to produce defined microvascular structures. Thus, the ability to induce site-specific microvessel network formation in vivo using minimally invasive ultrasound-based techniques has the potential to treat a range of clinical scenarios of tissue ischemia. Methods to create new microvascular networks in vivo are also critical to support implantation of engineered tissues that cannot rely upon passive diffusion to meet immediate metabolic demands. As well, future advances of this technology may offer exciting new approaches to engineer whole organs and tissues directly in vivo.

## Materials and methods

### USWF exposure apparatus

Transducers were driven by a dual-channel function generator (Tektronix AFG3022B, Beaverton, OR), attenuators (Kay Electronics Corp, Lincoln Park, NJ), and radio frequency (RF) power amplifiers (Electronics & Innovation, Ltd., Rochester, NY). Ultrasound fields were calibrated before and after each experiment using a membrane hydrophone (Marconi, bilaminar PVDF, Marconi Research Center, Chelmsford, England) and digital oscilloscope (Waverunner LT342, LeCroy Corp, Chestnut Ridge, NJ). Pressure amplitudes at 10.75 cm from each transducer were calibrated individually in the free field. Transaxial beam patterns of each transducer were measured independently. The − 6 dB beam width of each transducer was 1.2 cm at 10.75 cm from the transducer. To measure USWFs, two transducers were activated simultaneously and a membrane hydrophone was used to measure pressure amplitudes in the resulting USWF.

### Microparticle patterning in vitro

Sample holders were located in the USWF using a precision three-axis positioner system (Velmex, Inc., Bloomfield NY). Sephadex G-25 superfine microparticles (10–40 µm diameter; Pharmacia, Uppsala, Sweden) were mixed with 1% low melting-point agarose (w/v in degassed, deionized water) to a final concentration of 5 mg/mL, and pipetted into a sample holder. Sample holders were located in the USWF of the dual-transducer system using a precision three-axis positioner system (Velmex, Inc., Bloomfield NY). Two transducers were activated simultaneously in c.w. mode to generate an USWF at the location of intersecting beams. Samples were exposed for 10 min, during which time the agarose solidified. After exposure, the sample holder was removed from the water bath, and microparticle patterning was recorded using a Nikon digital camera equipped with a macro lens. Distances between planar bands of microparticles were quantified using ImageJ (National Institutes of Health) software. Transducer orientations of 180°, 120°, and 60° were tested.

### Cell culture and collagen gel preparation

Chemical reagents were from Sigma or ThermoFisher Scientific unless otherwise noted. GFP-HUVECs (Angio-Proteomie, Boston, MA) were used between passages 4–8. GFP-HUVECs were cultured in MCDB-131 basal medium (Gibco, Grand Island, NY) supplemented with 5% (v/v) fetal bovine serum (Hyclone, Logan, UT), 0.8% (v/v) ENDOGRO (VecTechnologies, Rensselaer, NY), and 2 mM l-glutamine (Gibco). Collagen solutions were prepared on ice by mixing 1 × Dulbecco’s Modified Eagle Medium (DMEM; Gibco) with 25 mM HEPES, 2 × DMEM, and type I collagen (rat tail; Corning) to produce final concentrations of 1 mg/mL collagen in 1X DMEM, pH 7.4. DMEM was degassed to minimize acoustic cavitation nuclei. Immediately prior to USWF exposure, GFP-HUVECs (1 × 10^6^ cells/mL), Sephadex particles (5 mg/mL), or cornstarch (5 mg/mL) were mixed with collagen solutions.

### In vivo acoustic patterning

C57BLKS/J mice (male, 10–16 weeks; Jackson Laboratory, Bar Harbor, ME) were used for non-recovery studies. C57BLKS/J mice were housed and maintained in a central animal care facility. Immunodeficient Rag1^null^ mice (B6.129S7-Rag1^tm1Mom^/J; male, 11–12 weeks; Jackson Laboratory strain #002216) were housed individually in sterile microisolator cages with sterile bedding. Rag1^null^ mice were provided autoclaved, acidified water and irradiated Modified ProLab^®^ Isopro chow containing 0.124% sulfadiazine and 0.025% trimethoprim (TestDiet). All animal procedures were reviewed and approved by the University Committee on Animal Resources at the University of Rochester. All experiments were performed in accordance with relevant institutional guidelines; the study is reported in accordance with ARRIVE guidelines.

Mice were administered buprenorphine (0.1 mg/kg) prior to procedures, and anesthetized using a combination of ketamine (80 mg/kg, Hospira Inc., Lake Forest, IL) and xylazine (8 mg/kg, AKORN Inc., Lake Forest, IL). Dorsal and ventral sides of mice were shaved and depilated using Nair^®^, and skin was cleaned with water. Needles (18-gauge, 1.5-in) were inserted ~ 5 mm below the right and left shoulders and guided subcutaneously down each side of the mouse such that needle tips were located ~ 10 mm above the hip (Fig. 2A, dotted arrows). To create space for collagen injections, a bolus of air (500 µL) was injected into each flank and then drawn out. Needles were left in place after removal of the air bolus. Mice were placed on a small animal holder attached to a three-axis positioner and lowered into the USWF exposure tank filled with 37 °C degassed, deionized water, with needle insertion sites located above the water surface. One injection site was positioned for exposure to the USWF, while the injection site on the contralateral side was located outside of the sound field to serve as a sham control. Collagen-cell or collagen-particle solutions (500 μL) were injected and needles were withdrawn. Transducers were activated for 3 or 10 min. Particle patterning was visualized in C57BLKS/J mice immediately after USWF exposure using high-frequency (38-MHz) ultrasound or confocal microscopy. Microvessel formation was assessed in Rag1^null^ mice 7 days after USWF exposure.

### High-frequency ultrasound and confocal imaging of patterned hydrogels

Mice (C57BLKS/J) were sacrificed immediately after USWF exposure and prior to ultrasound imaging to eliminate motion artifacts caused by breathing and heartbeat. Briefly, a broadband pulse, generated by a pulser-receiver (5073PR, Olympus, Waltham, MA), excited a 38-MHz, focused PVDF transducer (PI50-2, Olympus, Waltham, MA) at a pulse repetition frequency (PRF) of 1 kHz^37^. The transducer was focused at the center of each injection site using a three-axis positioner. The transducer was translated to generate images of transverse and sagittal cross-sectional planes. Imaging locations were separated by 1 beam width. A digital oscilloscope (Waverunner 62Xi-A, LeCroy Corp., Chestnut Ridge, NY) recorded ultrasound backscattered RF signals and B-scan images were generated, as described previously^10,37^.

Upon completion of high-frequency ultrasound imaging, USWF- and sham-exposed gels were excised and fixed with 4% paraformaldehyde. Gels were examined using an Olympus IX70 microscope equipped with a Disk Scanning Unit (Olympus, Canter Valley, PA) and SensiCam QE CCD camera (PCO-Tech Inc., Romulus MI). Images were obtained at 5-µm intervals and z-stack images were projected using MicroManager software (version 1.4, San Francisco, CA).

### Temperature measurements

Ultrasound-induced heating was measured using 50-µm copper-constantan thermocouples embedded in porcine muscle tissue samples (pork loin obtained from butcher, width ~ 4 cm). Measurements were performed ex vivo to provide worst case estimates, as convective and diffusive cooling from blood flow were absent. Thermocouples were inserted into tissue samples and positioned in sound fields using a three-axis positioner. Samples were exposed to 1-MHz, c.w. USWFs with peak pressure amplitudes of 0.3 MPa, 0.2 MPa, or 0.1 MPa generated by 2 transducers oriented at 180°. Temperature measurements were also made using pulsed USWF fields with peak pressure of 0.3 MPa and duty factors of 50%, 33%, and 25%. Measurements were taken using a digital laboratory thermometer (Physitemp Instruments, Model BAT-12, Clifton, NJ) sensitive to changes of 0.1 °C.

### Immunohistochemistry

To assess blood vessel formation by USWF-patterned human endothelial cells, Rag1^null^ mice (n = 6) were sacrificed 7 days after USWF- and sham-exposures. Injection sites and surrounding tissue, including underlying skeletal muscle, were excised and fixed in 10% neutral buffered formalin. Fixed tissues were embedded in paraffin, sectioned into 6-μm thick sections, and mounted on glass slides. Sections were chosen from the center of the injection area. Slides were incubated in antigen retrieval buffer (0.01 M sodium citrate, pH 6.0) at 60 °C for 90 min, then blocked with 3% bovine serum albumin (A3803, MilliporeSigma) and incubated overnight at 4 °C with rabbit anti-human Von Willebrand Factor (hVWF) monoclonal antibodies (D8L8G, Cell Signaling Technology; 1:200). After washing, bound antibodies were detected with SignalStain^®^ Boost IHC Detection Reagent (HRP, rabbit) and SignalStain^®^ DAB Substrate Kit (Cell Signaling Technology). Slides were counterstained with hematoxylin. Images were acquired using a Zeiss PALM^®^ MicroBeam Laser Capture Microdissection microscope and color camera. For each tissue block, a minimum of 6 sections spaced at least 60 μm apart were analyzed by 3 investigators blinded to exposure conditions.

### Supplementary Information

Supplementary Figures.

## Data Availability

All data are available in the main text or the supplementary materials.
