# Supplementary material for: In vivo acoustic patterning of endothelial cells for tissue vascularization
Source: Sci Rep. 2023 Sep 26;13:16082. doi: 10.1038/s41598-023-43299-0 (PMC10522665; doi:10.1038/s41598-023-43299-0)
Supplement: Supplementary file 1 — Supplementary Figures. [file 41598_2023_43299_MOESM1_ESM.pdf]

Supplementary Materials for

In Vivo Acoustic Patterning of Endothelial Cells for Tissue Vascularization

Eric S. Comeau *et al.*

Corresponding author email: dalecki@bme.rochester.edu

This file includes:

Figs. S1 to S2

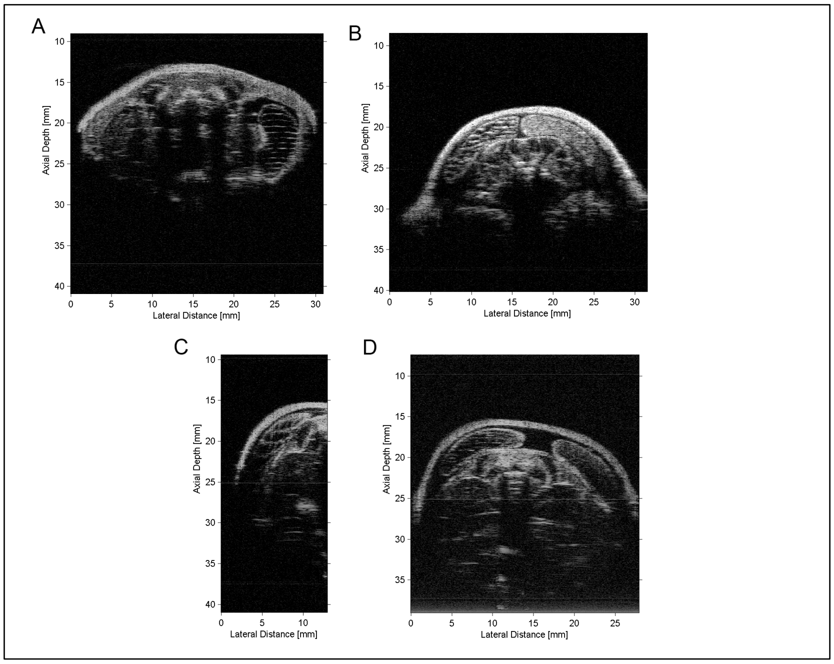

Fig. S1. High frequency ultrasound images of USWF-patterned microparticles in vivo. Solutions (500 μL) were injected into preformed subcutaneous skin pockets on either side of a mouse, and one site was exposed to an USWF. The contralateral injection site was located outside of the acoustic field. Representative high-frequency ultrasound images of a cross-sectional plane of a mouse post-exposure using the following USWF conditions: (A) cornstarch (5 mg/ml) in collagen (2 mg/ml) at 0.3 MPa, c.w., 180˚, 10 min. (B) cornstarch (10 mg/mL) and collagen (1 mg/mL) at 0.3 MPa, c.w., 60˚, 10 min. (C) Sephadex (7.5 mg/ml) in collagen (2 mg/mL) at 0.3 MPa, c.w., 180˚, 10 min. (D) Sephadex (7.5 mg/ml) in collagen (1 mg/ml) at 0.2 MPa, c.w., 180˚, 10 min.

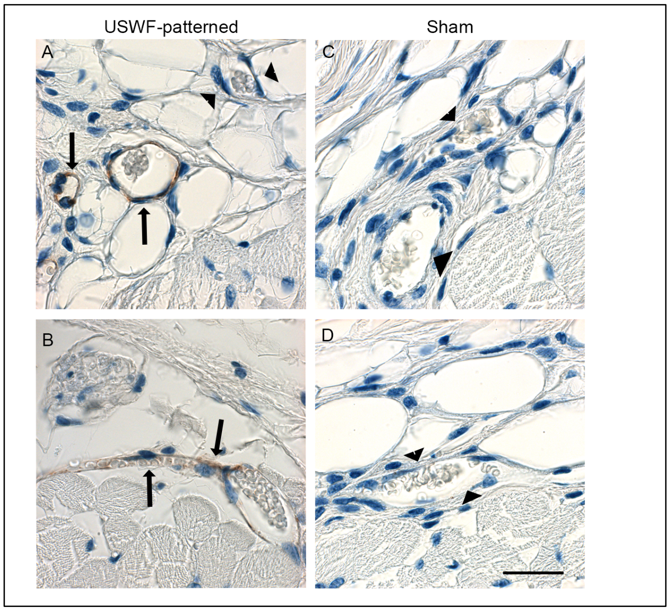

Fig. S2. hVWF-stained sections from USWF- and sham-exposed tissues. Solutions (0.5 mL) of HUVECs ($1x10^6$ cells/mL) and collagen (1 mg/mL) were injected into preformed subcutaneous pockets located on the flanks of $Rag1^{null}$ mice. One site was exposed to an USWF (1 MHz with peak amplitude of 0.3 MPa, c.w.) for 3 min. Seven days after acoustic patterning, mice were sacrificed and tissues corresponding to the injection sites were removed and processed for IHC. Human-derived endothelial cells were identified using an antibody specific to human VWF. Representative tissue sections from USWF- (A, B) and corresponding sham- (C, D) exposed regions. Arrows denote hVWF-positive blood vessels; arrow heads denote hVWF-negative blood vessels. Note that Panel A shows both hVWF-positive and hVWF-negative blood vessels in close proximity. Scale bar = 25 μm.
